# Septal Coronary Artery Injury After Left Bundle Branch Area Pacing

**DOI:** 10.1016/j.cjco.2024.12.005

**Published:** 2024-12-17

**Authors:** David Sánchez-Ortiz, Paula Vela-Martín, Daniel García-Rodríguez, Juan Francisco Oteo-Domínguez, Jorge Toquero-Ramos, Ignacio Fernández-Lozano, Víctor Castro-Urda

**Affiliations:** Cardiology Department, Hospital Universitario Puerta de Hierro Majadahonda, Madrid, Spain

**Physiologic stimulation therapies based on the capture of the left bundle branch of Hiss constitute a promising and expanding technique. A 61-year-old man with a history of ischemic heart disease with severe ventricular dysfunction underwent left branch pacemaker implantation. He presented chest pain immediately after the procedure, so coronariography was performed, showing dissection and obstruction of the fifth septal artery. The patient evolved correctly with conservative management. Given the widespread development and acceptance of the technique, an increasing number of possible complications are being described; although rare, they are directly related to the characteristics of the septal electrode implantation**

A 61-year-old man with a history of hypertension, dyslipidemia, and former smoking (ceased in 2005) was diagnosed with ischemic heart disease and severe ventricular dysfunction. In January 2023, he underwent drug-eluting stent revascularisation of the mid-left anterior descending artery. Follow-up revealed left bundle branch block with a QRS duration of 160 ms. Cardiac magnetic resonance imaging in May 2023 showed severe left ventricular dilation with an ejection fraction (LVEF) of 27%, and a nondilated right ventricle with mild dysfunction. Focal enhancements were noted at septal insertions with subtle intramyocardial striation.

In June 2023, the patient experienced decompensated heart failure requiring hospitalisation. Drug titration was challenging because of hypotension and mild renal function deterioration, leading to the decision to implant a cardiac resynchronisation therapy device. The procedure was complicated by venous coronary anatomy, necessitating conversion to left branch pacemaker implantation using a Biotronik Selectra 3D-55-39 catheter and a stylet-driven Solia S 60 electrode.

Preprocedural electrocardiography (ECG) showed left bundle branch block morphology with a QRS duration of 160 ms. Implantation parameters included a left ventricular activation time of 92 ms, right latency of 126 ms, threshold of 1 V, R-wave of 7, impedance of 617, nonfused QRS width of 149 ms, and fused QRS width of 116 ms with Qr morphology, indicating nonselective RI capture.

After the procedure, the patient experienced oppressive chest pain without repolarisation changes on the baseline ECG. Echocardiography was performed in the room, which ruled out the presence of pericardial effusion and showed no abnormalities compared with previous studies. Coronary angiography, performed because of suspected coronary lesion, revealed dissection and obstruction at the fifth septal artery. (Fig. 1). These findings were not present in the patient’s previous coronary angiograms. Conservative management was chosen owing to the vessel’s small calibre. After angiography, the pain was controlled with minimal analgesia, and high-sensitivity troponin I peaked at 42,120 ng/L. Follow-up showed LVEF improvement to 47% with 100% ventricular pacing.

**Figure 1:**
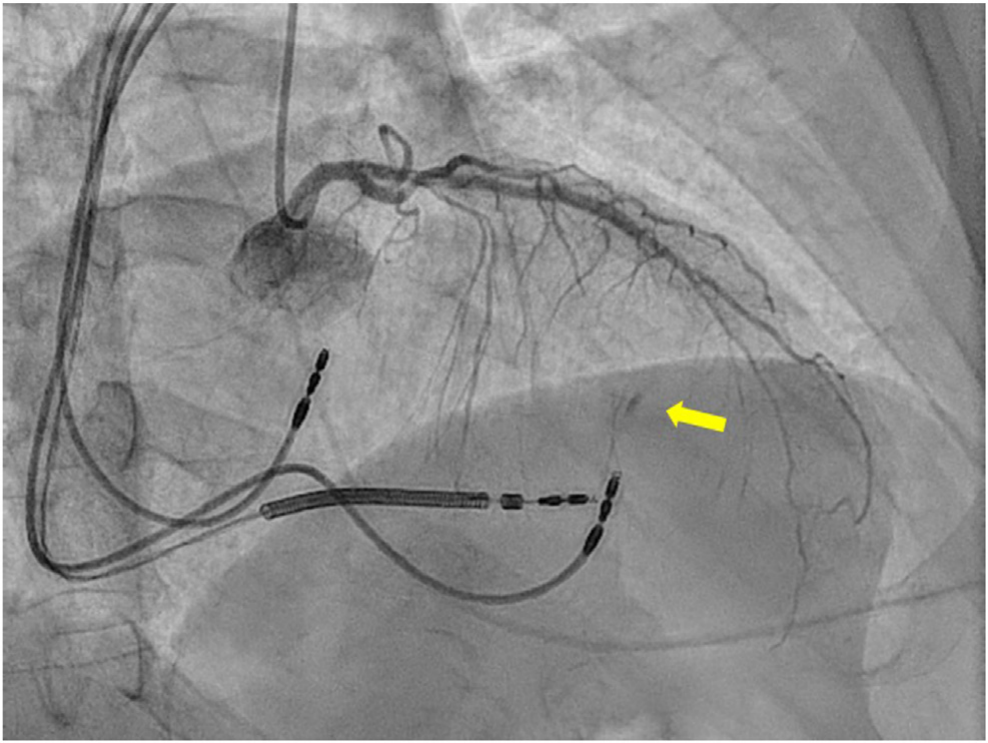
Coronary angiography, right anterior oblique 29° cranial 17° projection. Contrast extravasation is observed in the middle septum, probably dependent on a fifth or sixth septal branch. The filling is retrograde, by homocoronary flow. A **yellow arrow** highlights the lesion.

## Discussion

The presented case corresponds to one of the complications described as physiologic stimulations increase.^1^ Cases documented in the literature typically involve multiple attempts at septal perforation before achieving successful capture.^2^ Prospective registries indicate a slightly higher success rate with stylet-driven leads, but also a higher complication rate, including electrode displacement and septal perforation. Several cases have documented the occurrence of coronary events after septal electrode implantation, attributed to stimulation-induced spasm or irritation. To prevent injury to the anterior descending artery and its branches, excessively anterior implantation should generally be avoided. The MELOS study reported a 0.08% rate of coronary fistula in 2533 procedures.^1^

For small-sized arteries, conservative management is recommended. Various embolisation materials can be used for larger shunts with significant hemodynamic repercussions.^3^ Coronary events after electrode implantation in a septal position may result from induced spasm or irritation. Avoiding excessively anterior implantation can prevent damage to the anterior descending artery and its branches.^1^ The 9-segment fluoroscopic method has shown greater implantation success and reduced adverse events.^4^^,^^5^

In conclusion, as left bundle branch physiologic stimulation becomes more common, an increase in related complications is foreseeable. Improvements in implantation systems and careful anatomic evaluation can help to reduce these complications and facilitate early diagnosis and treatment. The present case highlights the importance of considering coronary lesions in the differential diagnosis of chest pain related to left bundle branch pacing device implantation.

**Novel Teaching Points**

• As left bundle branch physiologic stimulation becomes more common, an increase in related complications is foreseeable.
• Improvements in implantation systems and careful anatomic evaluation can help reduce these complications and facilitate early diagnosis and treatment.
• It’s important to consider coronary lesions in the differential diagnosis of chest pain related to left bundle branch pacing device implantation.
